# Indirect Reciprocity; A Field Experiment

**DOI:** 10.1371/journal.pone.0152076

**Published:** 2016-04-04

**Authors:** Jacobien van Apeldoorn, Arthur Schram

**Affiliations:** 1 University of Amsterdam, Amsterdam, the Netherlands; 2 CREED and Tinbergen Institute, Amsterdam School of Economics, University of Amsterdam, PO Box 15867, 1001, NJ, Amsterdam, The Netherlands; 3 Robert Schumann Center for Advanced Studies, European University Institute, Via Roccettini 9, I-50014, San Domenico die Fiesole (FI), Italy; Middlesex University London, UNITED KINGDOM

## Abstract

Indirect reciprocity involves cooperative acts towards strangers, either in response to their kindness to third parties (downstream) or after receiving kindness from others oneself (upstream). It is considered to be important for the evolution of cooperative behavior amongst humans. Though it has been widely studied theoretically, the empirical evidence of indirect reciprocity has thus far been limited and based solely on behavior in laboratory experiments. We provide evidence from an online environment where members can repeatedly ask and offer services to each other, free of charge. For the purpose of this study we created several new member profiles, which differ only in terms of their serving history. We then sent out a large number of service requests to different members from all over the world. We observe that a service request is more likely to be rewarded for those with a profile history of offering the service (to third parties) in the past. This provides clear evidence of (downstream) indirect reciprocity. We find no support for upstream indirect reciprocity (in this case, rewarding the service request after having previously received the service from third parties), however. Our evidence of downstream indirect reciprocity cannot be attributed to reputational effects concerning one’s trustworthiness as a service user.

## Introduction

In modern economies the increasing importance of online commerce has lead to a mushrooming of interactions amongst strangers. This leads to a growing need for mechanisms that govern such interactions. In particular, many transactions require mutual trust and cooperation to succeed. In this respect, interactions amongst strangers are no different than many other economic interactions; they heavily rely on implicit contracts [1]. This is especially the case when actions are taken sequentially and one actor incurs costs before obtaining the benefits. In long-lasting relationships, direct reciprocity in give-and-take interactions has long been established as a mechanism that supports cooperation [2]. Other mechanisms are needed to support cooperation amongst strangers, however [3]. The past 15 years have shown an increased awareness that indirect reciprocity may provide precisely such a mechanism. Strategies involving indirect reciprocity can evolve under evolutionary pressures and result in a cooperative steady state [4]. Potentially, this makes indirect reciprocity a strong economic force. Evidence of its importance stems from both theoretical analysis [5] and laboratory experiments [6,7]. To the best of our knowledge, there is no clear statistical evidence from the field, however. We fill this gap and provide data from a field experiment explicitly designed to test for the occurrence of indirect reciprocity in a natural field setting. Our results provide clear evidence of indirect reciprocity by humans in their natural habitat.

Whereas direct reciprocity involves two actors where one directly rewards (punishes) kind (unkind) actions by the other, indirect reciprocity involves a third party (S1 File; [11–32]). The three actors interact in either of two ways. First, in *upstream indirect reciprocity* an individual B who has been treated kindly (unkindly) by individual A reciprocates by being kind (unkind) to a third individual, C. In *downstream indirect reciprocity*, B reciprocates A because A was kind (unkind) to C in the past. Theoretically, both are considered to be important in the evolution of cooperation amongst humans [4,5] and laboratory experiments have shown that people behave in the way the theory predicts [7–10].

Our natural field experiment is conducted in an international online community with (at the time of the experiment) 5.5 million members in 97.000 cities worldwide. These members provide each other with a free but costly service when traveling. A traveler can request this service from all members that are able to offer it. S/he does so by sending a service request. If a service request is accepted, s/he receives the service without payment. Hence, the service provider endures a cost for the benefit of the traveler. All members can repeatedly be matched with different others, either as a provider or as a traveler. The service concerned is always the same. These characteristics make this community very suitable for studying indirect reciprocal behavior. More details about the community are in S2 File [33–35]. We note that this community prefers not to participate in academic research and is therefore not named in this paper. More information will be sent in private communication, upon request.

Downstream reciprocity predicts that the probability of having a service request accepted is higher for those who have previously provided to others, than for those who have not. This would confirm the laboratory findings and provide empirical field evidence in favor of the theory of indirect reciprocity. To study this prediction, we created several new profiles on the online community. Half of these profiles signals a history of providing the service to others (‘service profiles’), whereas the other half does not (‘neutral profiles’). In all other aspects, the profiles are identical. We varied these profiles across gender and nationality. The latter distinguished between Dutch and Israeli profiles. This gives four traveler types, and for each we created a serving history and a neutral history (see Methods). With these profiles, we sent a total of 189 service requests to different community members worldwide. Their responses were used in the analysis. We chose Israeli and Dutch nationalities for our travelers because both nations have a reputation for traveling a lot while the cultures are seen as very distinct. This allows us to check whether responses to service requests differ depending on the background of the traveler.

Our experiment was especially designed to study downstream reciprocal behavior. In particular, such behavior yields the hypothesis:

**Hypothesis 1** (downstream reciprocity)

A service request sent from a serving profile has a higher probability of being rewarded than a service request sent from a neutral profile.

Note that, strictly speaking, our design (see Methods) distinguishes between on the one hand being called a ‘good person’ with evidence to support this (service profiles) and on the other hand being called a ‘good person’ without such evidence (neutral profiles). The evidence that characterizes the service profiles is previous service to others. This directly yields the interpretation underlying hypothesis 1 that the difference between the two profiles is the past service provided.

Though limited, public information in the community also allows for studying upstream reciprocity. In particular, we are able to construct a proxy variable (based on the number of references from other providers) that indicates the extent to which the receivers of our service requests had themselves enjoyed services by others in the past. Upstream reciprocity predicts a positive correlation between this proxy and acceptance of our service request. Thus,

**Hypothesis 2** (upstream reciprocity)

A service request sent to provider X has a higher probability of being rewarded, the higher is the number of references from other providers on X’s profile.

## Results

Table 1 provides an overview of responses to our service requests. It shows the response rates, and the answer to the service request. A response can be ‘yes’, ‘maybe’ or ‘no’. The aggregate response rate across all requests is 47% and higher for requests sent from serving profiles than from neutral profiles. Moreover, Israeli profiles (55%) are responded to more often than Dutch profiles (42%) while differences between men (48%) and women (46%) are small (we provide statistical evidence of differences across profiles, below). One reason that the response rates may be somewhat low is that the webmaster after a while withdrew the profiles we created (see Methods). Given that we alternated the profiles of the requests sent, the time that the members had to respond was equal across profiles and this bias did therefore not affect our comparisons. More specifically, the average number of days that requests had been out when the profiles were deleted varies from 3.4 for Dutch men with a service profile to 4.9 for Israeli women with a service profile. The difference between service and neutral profiles within the same nationality/gender was 0.5 days for both Dutch and Israeli women, 0 for Israeli men and 0.6 for Dutch men. These differences are statistically insignificant.

**Table 1:** Data Overview.

	Dutch				Israeli				All	
	Female		Male		Female		Male			
	Serv.	Neutr.	Serv.	Neutr.	Serv.	Neutr.	Serv.	Neutr.	Serv.	Neutr.
**#requests**	30	30	28	25	19	19	19	19	96	93
**#responses**	16	6	14	11	11	12	10	9	51	38
**resp. rate**	53.3%	20.0%	50.0%	44.0%	57.9%	63.2%	52.6%	47.4%	53.1%	40.9%
**# yes**	8	1	7	3	4	5	8	3	27	12
**# maybe**	3	3	2	2	4	3	1	1	10	9
**% yes/req**	26.7%	3.3%	25.0%	12.0%	21.1%	26.3%	42.1%	15.8%	28.1%	12.9%
**% yes/resp**	50.0%	16.7%	50.0%	27.3%	36.4%	41.7%	80.0%	33.3%	52.9%	31.6%
**% no/resp**	31.3%	33.3%	35.7%	54.5%	27.3%	33.3%	10.0%	55.6%	27.5%	44.7%

*Notes*. Columns distinguish between the profile types created, “Serv.” = serving profile; “Neutr.” = neutral profile. In addition: “resp. rate” = #responses/#requests*100%; # yes is number of offers to provide the service; # maybe = number of responses that kept open the possibility of providing the service (but did not yet offer it); “% yes/req” = #yes/#requests*100%; “%yes/resp” = #yes/#responses*100%; “%no/resp” = (#responses–#yes–#maybe)/ #responses*100%. Any member that replied to be willing to provide the service for at least part of the time requested is reported as a ‘yes’. A member that replied that no service could be provided is reported as a ‘no’. Other answers, such as *“I don’t know yet”*, *“let me come back to you in a few days”* or *“Can you tell me a little bit more about yourself first*? *I might be able to serve you”*, are reported as a ‘maybe’. Only the first reply is reported, meaning that a ‘maybe’ can never turn into a ‘yes’ or a ‘no’.

Table 1 provides a first indication of (downstream) indirect reciprocity in our data. In aggregate, serving profiles receive 12.2%-points more responses, such responses are 21.3%-points more likely to offer the service (i.e., are ‘yes’) and are 17.2%-points less likely to reject outright (‘no’). A request sent out by a serving profile has a 28.1% probability of being accepted straight away, compared to only 12.9% for neutral profiles. This indicates that downstream reciprocity plays a role in this field environment. Third parties reward a past of helping others. These third parties are not only more inclined to respond, but also more willing to offer help.

For a more formal analysis we use probit regression models and test hypotheses 1 and 2. To start, column 2 of Table 2 investigates the determinants of service providers’ decisions on whether or not to respond to a service request.

**Table 2:** Determinants of Providers’ Decisions.

	Respond		Yes		Yes or Maybe	
serving profile	0.152	2.18**	0.250	2.69***	0.229	3.23***
*profile type*^*#*^						
Dutch man	0.126	1.11	0.084	0.84	0.001	0.03
Israeli woman	0.245	2.19**	0.070	0.52	0.076	1.09
Israeli man	0.142	1.36	0.285	2.88***	0.094	1.10
*provider characteristics*						
male	0.134	1.91*	–0.076	0.53	0.069	0.49
age/100	–0.558	0.41	–0.008	0.00	2.273	1.01
#providers’ references	–0.004	0.39	–0.021	1.48	–0.023	1.67*
#travelers’ references	–0.007	0.72	0.006	0.55	–0.003	0.33
#friends	–0.007	1.64	0.005	1.05	0.008	1.37
able to provide	–0.017	0.29	–0.000	0.01	0.038	0.36
days	0.031	1.48	0.020	0.67	0.069	1.80*
#observations	189		89		89	

*Notes*. The first number in a cell denotes the marginal effect of the variable depicted in the row, in a probit regression explaining the dependent variable distinguished by the column; the second number gives the corresponding z-value. We use robust standard errors clustered at the profile type (eight clusters).

“Respond” = 1 if the provider sent any response at all and 0, otherwise. “Yes” = 1 if, conditional on responding at all, the provider agreed to provide the service, and 0, otherwise. “Yes or Maybe” = 1 if, conditional on responding at all, the provider agreed to provide or kept the option open (i.e., s/he did not reject the request), and 0, otherwise. “Serving profile” = 1(0) if the request was from a serving (neutral) profile. “Profile type” is a set of dummy variables indicating the gender/country background of the profile that sent the request (marginal effects are relative to Dutch women). “Provider characteristics” are obtained from the profile of the community member to whom the request was sent. “#providers’ references” denotes the number of references left by other members that have previously provided the service to the member to whom we sent a service request. “#travelers’ references” denotes the number of references left by other members that have previously received the service from the member to whom we sent a service request. “#friends” denotes the number of friends on the profile of the member to whom we sent a service request. “able to provide” = 1 (0) if the profile indicates the availability to offer the service as “yes” (“maybe”) (recall that no request can be sent to a member indicating “no”). “Days” indicates the number of days between submission of the request and the day for which the service was requested.

*(**,***) denotes statistical significance at the 10(5-,1-)% level.

The results provide strong support for the observation that a request sent by a member who has previously provided the service to others is more likely to receive (any kind of) a response than an otherwise identical member without this history. This result does not depend on inclusion of provider characteristics in the regression. If these are dropped, the estimated marginal effect drops to 12.6%, but remains significant (z = 2.02**). It does require correcting for profile types, however: a regression with only the variable “service profile” yields a marginal effect of 12.3%, with z = 1.37(n.s.).

Hence a first indication of indirect reciprocity is observed: people are more likely to receive a response if they have helped third parties in the past. The results also show that Israeli woman are 24.5%-points more likely to receive a positive response than Dutch women; an effect that is statistically significant at the 5%-level. Other pairwise differences between profile types are not significant at the 10%-level. Exploring why Israeli women are more likely to receive a response is beyond the scope of this paper, however.

Columns 3 and 4 of Table 2 consider the probability of receiving a positive response, conditional on receiving any response at all. They differ in how they treat the response ‘maybe’. In column 3, this is considered a rejection of the request and in column 4 we treat it as a positive reply. The marginal effects show that, conditional on receiving any response at all, the probability of receiving help is 25.0%-points (column 3) or 22.9%-points (column 4) higher if it was sent by someone with a history of helping others than if it was sent by someone with otherwise the same characteristics and reputation, but without the helping history. This result does not depend on including other independent variables. Regressions with only “service profile” yield marginal effects of 21.4% (z = 2.58***) for column 3 and 17.3% (z = 2.31**) for column 4.

The other result that stands out is that Israeli men are 28.5%-points more likely to receive a “yes” in response to their request than Dutch women (significant at the 1%-level). The 20.1%-point difference with Dutch men is also statistically significant (at the 5%-level). Once again, it is beyond the scope of this paper to explain such differences across profile types. Finally, note that the distinct responses to the two profiles rule out other-regarding preferences as the sole explanation for member’s willingness to provide the service to strangers. We know of no model of social preferences that would make this distinction. One could, of course, see indirect reciprocity itself to be a model of social preferences. We consider it more a behavioral strategy. Moreover, the fact that standard models of indirect reciprocity do not predict the patterns that we observe does not imply that they play no role at all. The fact that all member types at least sometimes receive “yes” as an answer may be an indication of prosociality.

The results for the “serving profile” variable in all three regressions provide strong statistical support for hypothesis 1 and are therefore evidence from the field of downstream indirect reciprocity. In fact, our data provide evidence if such indirect reciprocity for each of the gender/nationality types. If we interact the serving profile with profile type, significant differences between service profiles and neutral profiles (in favor of the service profiles) are found for each of the profile types except Israeli women, where differences are statistically insignificant. Note that such a split in subgroups drastically reduces the numbers of observations, however.

Of course one can also combine the response decision of column 2 with either of the decisions in columns 3 and 4 (i.e., classify non responses as a ‘no’). This gives further support to the hypothesis: for columns 2/3 combined the marginal effect of having a serving profile is estimated to be 0.175 (z = 3.42***) and for 2/4 it is 0.195 (z = 3.50***). This shows that also the unconditional probability of receiving help is significantly higher for serving profiles than for neutral profiles. Similar support is obtained if the response “maybe” is treated as a separate variable and an ordered probit regression is conducted (details are available upon request).

To evaluate hypothesis 2 (upstream reciprocity) we consider a variable that measures the number of references left by service providers on the profiles of the members to whom we sent a service request (“#providers’ references” in Table 2). Recall that this is used as a proxy for a member’s past traveling behavior. A positive coefficient for this variable would indicate that the willingness to respond positively to our service request is increasing with the number of times that a member has received service from others in the past, i.e., it would provide evidence of upstream reciprocity. Our results show no such effect. In fact, all three coefficients are negative, one of them significantly so at the 10%-level. One possible reason is that members with many references left by service providers tend to use the community to receive services and are less inclined to offer them. Notice that this is in sharp contrast to upstream reciprocity.

## Discussion

Our results provide first solid evidence from the field of downstream indirect reciprocity. Keeping all other characteristics equal (including the reputation of being ‘kind’), we have shown that a history of helping strongly increases the probability of a positive response to a request for help. This probability was unrelated to the amount of help previously received by the person to whom the request was sent, however. We thus found no support for upstream indirect reciprocity.

This evidence from the field has important implications for understanding cooperative behavior. It confirms previous laboratory findings and provides further support to the idea developed in theoretical biology that indirect reciprocity is a mechanism that supports cooperation amongst strangers. This suggests that indirect reciprocity may be important in establishing trustworthiness in transactions that involve incomplete contracts. It implies, for example, that an individual engaged in a transaction with a stranger is more likely to be treated fairly if she herself has a history of acting fairly in trades with strangers. If indirect reciprocity does play this role, then this points to institutions that will help in fostering further cooperation. In particular, an individual A, deciding on whether to act cooperatively to some other person B, would require a reputation mechanism that specifically indicates B’s previous behavior in situations comparable to A’s current decision.

Note that the information about an individual’s reputation that is needed to enable indirect reciprocity is much more specific than, e.g., a reputation indicating what kind of person B is. In that respect, information about the individuals in our serving profiles was the same as in our neutral profiles. It is conceivable, of course, that information from the neutral profiles is considered to be more reliable than information from the serving profiles (e.g., because it is from people who have allegedly ‘known’ the person concerned much longer) or vice versa. We purposely phrased the references such that they are appear more credible coming from a ‘friend’ than from someone met only for a few days (e.g., “… is a very good person”). This ensures that any potential bias would decrease the likelihood of observing indirect reciprocity.

The information needed is also not about previous choices an individual made when in the same situation as now. The latter could be used to update the probability about how this individual will act in the current transaction. In our design, this would be possible if we added references from other service providers to our profiles, our profile being the service recipient. The member to whom we sent a service request could use these references to judge how the traveler would behave if our request were granted. Because this would interfere with the information about previous behavior of our profile as a service provider (which is needed to enable indirect reciprocity), we chose not to add such service references. This allowed us to isolate the effects of information about the history of service provision.

Note that we do not address the mechanisms underlying indirect reciprocity. One possibility (suggested by an anonymous reviewer) is that service providers *trust* more a request from someone with a history of offering the service than someone without this history. Investigating such mechanisms is beyond the scope of this paper. In the case of trust, for example, it would require understanding how trust in someone’s behavior as a service user relates to their behavior as a provider.

An interesting next step would be to investigate various reputation mechanisms in the field to study the effects of distinct information about individuals’ history of helping on the development of indirect reciprocity. One can think of variations in the length of history; mixtures of information about on the one hand direct encounters between two parties and on the other a history concerning third parties; second-order information about why someone did or did not help strangers in the past (which would allow for so-called ‘standing strategies’ [10, 36]; etc. A different path of research could investigate further the reasons for the lack of upstream reciprocity in our field setting. Though such responses to one’s own history are thought to be important in the evolution of cooperation [4], our data show no evidence at all that humans behave in this way. It would be interesting to investigate whether there are environments more favorable to upstream reciprocity than the online community that we have investigated.

## Methods

For each of the four gender/nationality cells we created two profiles, ‘serving’ and ‘neutral’. To each profile, we added self-reported experience and a set of 10 references from ‘other’ users. On the serving profiles, we formulated the self-stated experience as follows:

*“I’ve only [provided service] so far*. *I love to meet different people this way and exchange information and experiences about our cities and cultures*.*”*

(Throughout this paper, in order to avoid revealing the online community, we replace identifying phrases by neutral terms in square brackets ([…]).)

On the neutral profiles, it reads almost exactly the same:

*“I have no […] experience yet*. *I’d love to meet different people this way and exchange information and experiences about our cities and cultures*.*”*

The ten references were created by asking ten existing members to participate in the experiment. They posted these references (designed by us) on the created profiles. These members were aware of the purpose of the experiment. They were also carefully instructed on what reference to leave on which profile. All serving profiles were given references from travelers and all neutral profiles received neutral references. No profile was given the same reference more than once and no reference was written by the same person more than once (not even on different profiles; since references for other members are displayed on a profile, it might be suspicious if a member left identical references on more than one profile). All serving (neutral) profiles were given exactly the same ten references. Note that the latter will not affect service providers’ decisions, because each received a request from only one profile.

Participating members made no mistakes in following the instructions. The process thus yielded twenty distinct references, ten of which were written on behalf of a ‘traveler’ and ten in the name of a ‘neutral friend’, i.e. by someone claiming no interaction as a member. The two sets of ten references were paired, with the same words used within each pair. For example, one of the references left by a traveler is:

“*Peter is a very good [provider]*. *He is welcoming*, *knows a lot about Amsterdam and is fun to hang out with*.*”*

The neutral reference of this pair is:

“*Daniel is a very good person*. *He is welcoming*, *knows a lot about Amsterdam and is fun to hang out with*.*”*

The ten reference pairs used are given in S3 File. All serving profiles received the first reference of a pair and all neutral profiles received the second. In this way, the serving profiles are given the same positive reputation as the neutral profiles, with the only difference being that their references also signal that they have provided the service to others in the past, which is not the case for the neutral profiles.

Other than these signals about past provision, the serving profiles do not differ from the neutral profiles (see S4 File for an overview of all text written on the profiles). One exception is the profile picture. Since the community regulations do not allow duplicate profiles or fake identities, real identities had to be used. Eight individuals (four men, four women, crossed with four Israeli and four Dutch) who were not yet a member were asked to participate in this experiment by giving permission to use their real name and picture to create a profile. All pictures were taken from a distance, minimizing the possible effects of appearance (see S5 File for the pictures that were used; the individuals concerned have given written informed consent to publish these pictures). There were two individuals in each of the gender/nationality combination, one was randomly assigned to a serving profile, the other received a neutral profile. Of course, we cannot exclude the possibility that the pictures convey information that we do not control and that this could explain some of the behavior we observe. Note that the fact that pictures were randomly distributed across the two profiles diminishes this problem.

All profiles were used to randomly send out a large number of service requests to different members worldwide. Note that this procedure involves deception of the members who receive a request. The non-deception rule that is applied to laboratory experiments is typically not upheld for field experiments, however (for an example of a well-cited field experiment involving deception, see [37]). There are many reasons for this distinction between the laboratory and the field. The most obvious is that participants in natural field experiments like ours do not know that they are part of an experiment. There is little danger that they will detect the deception and respond to it. Similarly, the chance that this deception (even after debriefing) will affect behavior in subsequent experiments is negligible. The possibility of an (uncontrolled) response to perceived deception in an ongoing or in future experiment(s) is the main reason why economists have effectively banned deception from laboratory experiments.

Selection of the members that received a request was randomized over a restricted subset of all community members. In particular, only members that had a status denoting that their availability to offer the service was ‘yes’ or ‘maybe’ could be sent a service request. As a result, only these members could be selected. A second restriction, imposed by us, is that the last time a member had logged in, was no longer than two weeks prior to the selection. This was done to increase the probability that the requests would be read within a reasonable time frame. Under these two restrictions, 189 members were randomly selected and each was randomly allocated to receive a request from either a service profile or from a neutral profile (with equal probabilities). This ensures that possible treatment effects are not affected by specific characteristics of the members concerned (see S6 File for statistical evidence). Requests were sent between May 8 and May 13, 2013 for service provision starting May 22^nd^. We originally considered sending out more requests later. On May 14, the eight experimental profiles were deleted by the community webmaster, however, for using the community for other purposes than is intended. Because we had alternated the requests sent across profiles, at the time of removal sufficient data had been collected for all profiles to test our hypotheses. Also, after all data had been collected, we debriefed all providers that we had approached by sending an email that briefly explained the project and their role therein. Not one objected to this.

The service request sent is exactly the same for serving profiles and neutral profiles, except that the serving profiles again signal their history of service provision, whereas the neutral profiles signal their neutral history. The requests are displayed in S7 File. When sending a service request, there are several fields that need to be filled out. Amongst other things, one has to indicate “why I would like to meet you”. This field is mandatory and the request cannot be sent if fewer than 100 characters have been written. It is suggested that one provides personal comments that show that one has actually read the provider’s profile. Since it could seem suspicious if nothing personal were written here, this field could not be identical for all requests. However, to keep the messages alike among all requests, one specific sentence is used, with some words adapted to refer to the provider’s profile. The sentence that was used reads as follows:

*“You seem like a really nice person and some statements on your profile like………….. and…………..and…………….sound like me*!*”*

Examples of what could be written on the dots are *“that you are into sports and modern art”* or *“that your philosophy is to live day by day”*. Thus, the requests were written such that all approached providers read the same basic message and the same personal sentence with three elements that referred to their own profile. Again, the only difference across requests is that serving profiles signal a history of helping others, whereas neutral profiles do not.

Table 3 shows how the 189 requests were distributed across the eight experimental profiles. 96 requests were sent from serving profiles and 93 from neutral profiles. Also within each category, the number of requests sent is balanced between the serving and neutral profiles.

**Table 3:** Service requests sent.

		Female & Dutch	Male & Dutch	Female & Israëli	Male & Israëli	Total
History type:	Provision	30	28	19	19	96
	Neutral	30	25	19	19	93
	Total	60	53	38	38	189

*Notes*. Cells show per profile the number of requests were been sent. S8 File provides the raw data set acquired from these requests.

As mentioned above, this design was never intended to be used to investigate upstream reciprocity. Nevertheless, there are some indicators that reveal a members’ past in terms of traveling, which allow us to develop a proxy that indicates this history. This proxy is determined by the number of references left by previous service providers on the profile of the member who has been approached with a request. Such a reference indicates that service has been received from this referee. To test for upstream reciprocity, we investigate the correlation between this proxy and the probability that a service request sent to this member will be rewarded.

### IRB approval

The ethics committee of the Dept of Economics and Business of the University of Amsterdam approved the field experiments with human subjects reported in this study. The IRB, chaired by prof. dr. J. Sonnemans, granted approval based on the observation that the experiment adheres to the rules set by the Center for Research in Experimental Economics and political Decision making (CREED). No specific approval for specific experiments, such as ours, is required. CREED is a renowned institute for experimental economic research and adheres to the standards set in experimental economics. The collection, storage, protection, retention, and destruction of all data comply with national and EU regulations. All users of the online platform that were approached for this experiment were debriefed via email about the study's methods and goals (see Methods). The individuals depicted by photograph in S5 file have given written informed consent (as outlined in PLOS consent form) to publish these pictures.

## Supporting Information

S1 File Direct and Indirect Reciprocity. (PDF)

S2 File The Online Community. (PDF)

S3 File References Used. (PDF)

S4 File Texts on Created Profiles. (PDF)

S5 File Pictures Used. (PDF)

S6 File Member Characteristics. (PDF)

S7 File Service Requests Sent. (PDF)

S8 File Raw Data. (XLSX)
