# Supplementary material for: Indirect Reciprocity; A Field Experiment
Source: PLoS One. 2016 Apr 4;11(4):e0152076. doi: 10.1371/journal.pone.0152076 (PMC4820101; doi:10.1371/journal.pone.0152076)
Supplement: S1 File — (PDF) [file pone.0152076.s001.pdf]

Direct and Indirect Reciprocity

Originally, direct reciprocity was discussed mainly in other disciplines than economics [11-15]. Following the rise in Experimental Economics in the 1980s, economists started to recognize the importance of direct reciprocity in many economic interactions. Laboratory control enabled a careful isolation of this behavior in a variety of games. These include the investment game [16-17], prisoners' dilemma [18-20], gift exchange game [21] and public good games [22,23]. Most experimental studies reporting directly reciprocal behavior utilize data from the laboratory [2,17,19,21,23-25]. Further support comes from the field [26].

Early on, the biologist Trivers noted the possibility of a 'generalised altruism', where "individuals …. may respond to an altruistic act that benefits themselves by acting altruistically toward a third individual uninvolved in the initial interaction" [12, p53]. Later, Alexander [27] introduced the term 'indirect reciprocity' and stressed that reputations provide the links between individuals that can allow indirect reciprocity to work. Agent-based simulations of strategies that allow individuals to condition their cooperative behavior on a partner's reputation ('image score') show that indirectly reciprocal strategies can be evolutionarily stable [5]. One of the driving forces is that having a good reputation may pay if it invokes future cooperative behavior by third parties. This theoretical result is generally considered to be a milestone in understanding the evolution of human cooperation. Though the specific results found by Nowak and Sigmund have been argued to crucially depend on the set of strategies allowed [28], it is now widely believed that indirect reciprocity plays a key role in human cooperation [4, 29].

Early laboratory evidence of indirect reciprocity is provided by [30] who detect downstream reciprocity in an ultimatum game where subjects were given information about their partners' previous choices vis-à-vis a third party. Other experiments were designed after Nowak and Sigmund [5] had established the evolutionary importance of indirect reciprocity. Evidence shows that people invest more in a public good game after they learn that the recipient has recently made a donation to a charitable institution [31]. Such downstream reciprocity has also been observed in the investment game [17,32] and the repeated helping game[1] [3,7,9]. In the repeated helping game, a large group of individuals is repeatedly and anonymously matched into donor-recipient pairs. Donors decide whether or not to incur a cost c to give the recipient a benefit b, with $b>c$. To study the occurrence of indirect reciprocity, donors are given information about (some of) the recipient's previous decisions when they were in the role of donor. For more details, see [7]. Finally, upstream reciprocity is found by [8] where subjects grouped in cyclical networks participated in an investment game [16].

All in all, there is a rich theoretical literature on the important role that reputation and indirect reciprocity play in the evolution of human cooperation, and there is ample evidence from laboratory experiments with human subjects that individuals use indirectly reciprocal strategies. However, evidence using natural data from a controlled field setting is still missing. Without such data, the external validity of previous results remains unclear.

---

[1] I
