# Supplementary material for: Indirect Reciprocity; A Field Experiment
Source: PLoS One. 2016 Apr 4;11(4):e0152076. doi: 10.1371/journal.pone.0152076 (PMC4820101; doi:10.1371/journal.pone.0152076)
Supplement: S2 File — (PDF) [file pone.0152076.s002.pdf]

The Online Community

The community we use for our experiment offers a field environment that is characterized by all the elements necessary to enable indirect reciprocity. In particular, members are asked to endure costs to offer a service without any benefits other than a gain in reputation that is (only) relevant within the community. All 5.5 million members have their own profile. These profiles are public and contain basic personal information, pictures and references left by other members. A traveler can request the service from any member that is able to provide it, by sending out a service request to such a member. A request can be answered with 'yes', 'maybe' or 'no'. An accepted request is an offer to provide the service free of charge. The provider can thus decide whether or not to help the traveler. When helping, the provider endures costs in terms of time, effort, and in many cases money. Typically, these costs are lower than the benefit for the traveler. Members repeatedly interact, either as a traveler or as a provider. All of these characteristics fulfill the standard assumptions of the repeated helping game [5], in which laboratory subjects show clear evidence of indirect reciprocity [7].

Since providing the service to other members is not in one's direct monetary self-interest, standard assumptions in economic theory applied to self-interested decision makers predict that people would do no such thing. Of course, service providers may also derive utility from the interaction with a traveler. This does not interfere with the possibility of indirect reciprocity, however. Moreover, there are economic theories outside of the mainstream that could be adapted to account for service provision to strangers. Think of models of community enforcement [33,34] or models of social preferences [35].

The website is an enormous success and the number of total services offered exceeds 5 million. Furthermore, most interactions are indirect, meaning that traveler and provider usually meet each other only once. Direct reciprocity can therefore not explain the observed activity either. It is conceivable that providers offer the service because of other-regarding preferences. Our results allow us to reject this explanation of the choices observed in our experiment, however.

Apparently some other mechanism is driving cooperation in this community. Because the benefits of receiving the service exceed the costs of providing it, it can be beneficial to provide the service to others, if this increases the probability of receiving the service as a traveler sometime in the future. This is the case if having a reputation of providing the service increases the probability that a provider will offer the service to you later on; in other words, if indirect reciprocity is involved. Note that it is not a reputation of being a good person in general or a trustworthy community member that drives indirect reciprocity. Instead, the reputation is about having served others in the past. Our design allows us to explicitly isolate the latter type of reputation.

It follows that in order to condition on somebody's serving history, information about this history is needed. In this community a member's profile contains two indicators that reveal her history in terms of providing and receiving the service. The first is a self-provided indication of her experience in both respects. Because there is no check on the truth of this statement, the second indicator is more reliable. It uses references that are left on the profile after an interaction has taken place. On every profile, all references are displayed that have been received from or written to other members. Importantly, the profile also shows whether a reference was left by a provider, a traveler or by a 'neutral' party. A neutral party was not involved in a service interaction with the member concerned but can be a friend that is also a member, for example to inform the community that the person concerned is reliable and trustworthy. This results in a better reputation, but not in terms of a history of service provision. Only a reference left by a traveler signals that a member has provided the service in the past and thus affects one's reputation in this respect. The number of references left by travelers is therefore a good indicator of how much help has been offered in the past. This, together with a member's stated experience, is the available information about a member's history. Information on the number of times service was denied is not available.

In sum, members repeatedly make choices on whether or not to help other members, where the cost of helping is lower than the benefit of receiving help. There is little room for direct reciprocity, since most members interact only once with the same partner. All members can be either a provider or a traveler. And lastly, when deciding on whether or not to help a certain member, information is available that reveals this member's past in terms of helping others. Because of these characteristics, this community is perfectly suited to study indirect reciprocity in a field setting.
