# Supplementary material for: Indirect Reciprocity; A Field Experiment
Source: PLoS One. 2016 Apr 4;11(4):e0152076. doi: 10.1371/journal.pone.0152076 (PMC4820101; doi:10.1371/journal.pone.0152076)
Supplement: S3 File — (PDF) [file pone.0152076.s003.pdf]

## References Used

In this appendix, phrases in the references that identify the specific online community used have been replaced by neutral phrases in […]. The complete original references are available from the authors, for private investigation. All references are given in pairs, below. An existing member was asked to leave a reference at a profile we created; either as a previous service recipient or as a friend. The response we gave from the profiles is given below the reference. Words in {} were adjusted to fit the specific person we created.

| Pair | reference from service recipient<br>*response from 'our' serving profile* | reference from friend<br>*response from 'our' neutral profile* |
|---|---|---|
| 1 | I [used {name}'s service] and I had a great time. {He/she} told me a lot about the city, which was very interesting. {name} is a good [service provider]!<br><br>*We had a lot of fun together and I really enjoyed [providing the service to {name}] !* | {Name} is a good person, we always have a great time. He/she can tell a lot about {city} and is very interesting.<br><br>*{name} is a very nice person and a lot of fun to hang out with.* |
| 2 | We had a lot of fun getting to know each other and talking about our cities and cultures. Good [service provider], nice {guy/girl} and definitely recommendable!<br><br>*{name} was a nice [service recipient], I'd recommend {him/her} to other [members of the community]!* | {Name} and I are friends and always have a lot of fun together, I would definitely recommend him/her to other *[members of the community]*!<br><br>*{name} is a good friend and I'd recommend him/her to other [members of the community] as well!* |
| 3 | {Name} is a very good [service provider]. {He/she} is welcoming, knows a lot about {city} and is fun to hang out with.<br><br>*It was definitely worth getting to know {name}. [Providing the service to] {him/her} has been great.* | {Name} is a very good person. {He/she} is welcoming, knows a lot about (city) and is fun to hang out with.<br><br>*{name} and I always have a lot of fun hanging out. {He/She} is a very good person and I'm sure it'll be fun [Providing the service to] {him/her.}* |
| 4 | Very open, interesting, fun person to hang out with. {He/she} has been a very good service provider] to me.<br><br>*{name} was a great guest. Very friendly, interesting and open minded.* | Very open, interesting, fun person to hang out with. {He/she} is a good friend of mine.<br><br>*{name} and I are friends and I'd say{ he/she} is a person that is definitely worth getting to know, so if you [provide the service to] {him/her} I'd say do it!* |
| 5 | I [received the service from] {name} and it was great getting to know each other. Whenever {name} had time, {he/she} would explore the city with me and show me nice places where you don't usually come as a tourist.<br><br>*It was interesting and fun showing {name} around in the city. We had a great time.* | It's great to know {name} and to hang out with {him/her}. {He/she} can also show [travelers] places where you don't usually come as a tourist.<br><br>*{name} would for sure be a great [service provider or traveler], {he/she} is a very nice and fun person.* |
| 6 | Nice {guy/girl}, good [service provider]. I had a good time using {name's} service]!<br><br>*Very friendly, enthusiastic {girl/guy} and a good [service recipient]!* | Nice {guy/girl}, good friend. I think you'll have a good time [using the service of] {name} or [providing the service to {him/her}.<br><br>*{name} is a good friend of mine and I would definitely recommend her/him to all [members], because I'm sure you'll have a good time with {her/him}!* |
| 7 | I [used {name's} service] and I had a great time. It was a very pleasant [experience] and I feel like I experienced {city} in a way I otherwise wouldn't have. {Name} was a great [service provider]!<br><br>*{Name} and I had fun exploring {city} together and getting to know each other!* | {Name} is a great friend and we always have a great time! {He/she} can give you an experience of (city) that you'd otherwise not have.<br><br>*{Name} and I have been friends for a while and {he/she} is a great person, friendly and open.* |
| 8 | I'd say {name} is a very good [service provider], who makes you feel very welcome. It was a very pleasant [travel] experience and I really liked {city} too. | {Name} is very welcoming and a very good friend. Always pleasant experiences in {city} with him/her. |

| | | |
|---|---|---|
| | *{Name} has been a great [service recipient], I enjoyed [providing the service to] {him/her} a lot.* | *I'm sure [providing the service to] or [receiving the service from] {name} is a great experience, {he/she} is a good friend of mine.* |
| 9 | {Name} and I had a good click and we had fun exploring {city} and learning from each other's cultures. {He/she} is a very nice, interesting and fun person to hang around with!<br><br>*{Name} is very open for other cultures and it was a great experience showing {him/her} around here and getting to know {him/her}!* | {He/she} is a very nice, interesting and fun person to hang around with! We have a good click and it's fun to learn from each other.<br><br>*Always a good time with {name} and good conversations!* |
| 10 | I asked {name} pretty last minute to [provide the service to] me and {he/she} was very helpful, welcoming and friendly. I'm really glad I [received the service from] {name} cause it made my {city}'s experience a lot more exciting and fun!<br><br>*It was my pleasure [providing the service to] {name}. Glad I could help and get to know you!* | {Name} is very helpful, welcoming and friendly. Hanging out together is always fun and exciting.<br><br>*{Name} is a good friend and we always have a good time together.* |
