# Supplementary material for: Indirect Reciprocity; A Field Experiment
Source: PLoS One. 2016 Apr 4;11(4):e0152076. doi: 10.1371/journal.pone.0152076 (PMC4820101; doi:10.1371/journal.pone.0152076)
Supplement: S4 File — (PDF) [file pone.0152076.s004.pdf]

Text on the Created Profiles.

This appendix provides an overview of the texts entered on the profiles that we created on the online community. References that identify the specific online community used have again been replaced by neutral phrases in […]. Again, precise texts used are available upon request in private correspondence.

Personal Description
I would describe myself as being friendly, easy-going and positive. I like traveling and sports. I'm still in university at the moment, but will start working next year hopefully.

How I Participate in [this community]
For serving profiles:
I've only [provided the service] so far. I love to meet different people this way and exchange information and experiences about our cities and cultures.
For neutral profiles:
I have no [traveling] experience yet. I'd love to meet different people this way and exchange information and experiences about our cities and cultures.

Interests
I like sports, traveling and reading.

One Amazing Thing I've Seen or Done
I saw the Grand Canyon, that was amazing.

Opinion on the [online community] Project
I really like the idea!
