# Supplementary material for: Indirect Reciprocity; A Field Experiment
Source: PLoS One. 2016 Apr 4;11(4):e0152076. doi: 10.1371/journal.pone.0152076 (PMC4820101; doi:10.1371/journal.pone.0152076)
Supplement: S5 File — (PDF) [file pone.0152076.s005.pdf]

Pictures used for the Profiles.

	Female & Dutch	Male & Dutch	Female & Israeli	Male & Israeli
**Serving profile**	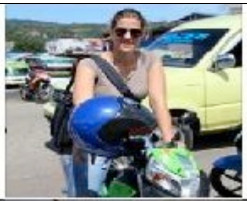	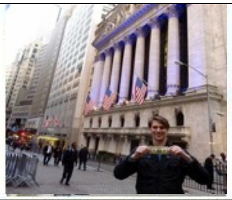	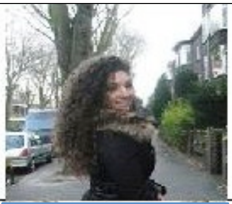	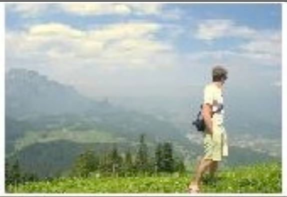
**Neutral profile**	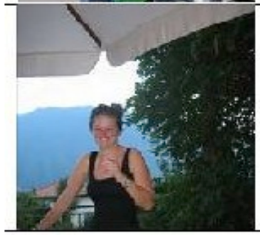	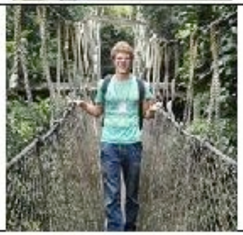		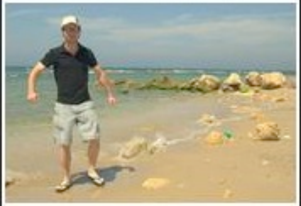

*We were unable to obtain consent to use the picture of the female Israeli assigned with a neutral profile in a publication.
