# Supplementary material for: Indirect Reciprocity; A Field Experiment
Source: PLoS One. 2016 Apr 4;11(4):e0152076. doi: 10.1371/journal.pone.0152076 (PMC4820101; doi:10.1371/journal.pone.0152076)
Supplement: S6 File — (PDF) [file pone.0152076.s006.pdf]

Member Characteristics

This appendix provides evidence that characteristics of members that were selected to receive a service request, are uncorrelated with the type of profile that was used to send the request. To test this, we ran a probit regression of the profile type (a dummy variable equal to 1(0) for a serving (neutral) profile) on the available characteristics of the members that are selected to receive a request. Table S6-1 gives the marginal effects derived from this regression.

Table S6-1: Probit Regression

	Serving Profile	
male	0.028	0.34
age/100	0.006	0.54
#providers' references	0.001	0.09
#travelers' references	0.011	1.21
#friends	–0.008	1.75*
able to provide	–0.055	0.70
days	0.002	0.07
#observations	189	

Notes. The first number in a cell denotes the marginal effect derived from the probit regression of the variable depicted in the row; the second number gives the corresponding z-value. "Serving profile" = 1(0) if the request was sent from a serving (neutral) profile. The gender and age refer to the member who received the request. "#providers' references" denotes the number of references left by other members that have previously provided the service to the member to whom we sent a service request. "#travelers' references" denotes the number of references left by other members that have previously received the service from the member to whom we sent a service request. "#friends" denotes the number of friends on the profile of the member to whom we sent a service request. "able to provide" = 1 (0) if the profile indicates the availability to offer the service as "yes" ("maybe") (recall that no request can be sent to a member indicating "no"). "Days" indicates the number of days between submission of the request and the day for which the service was requested.
*denotes statistical significance at the 10% level.

The results show very small effects of members' characteristics on whether the request is received from a serving profile or a neutral profile. One out of the seven independent variables is statistically significant at the 10%-level, but even here the marginal effect is very small (0.8 %-points).
