# Supplementary material for: Indirect Reciprocity; A Field Experiment
Source: PLoS One. 2016 Apr 4;11(4):e0152076. doi: 10.1371/journal.pone.0152076 (PMC4820101; doi:10.1371/journal.pone.0152076)
Supplement: S7 File — (PDF) [file pone.0152076.s007.pdf]

Service Requests Sent

The following service requests were sent from the two profile types. As before, we replace phrases that identify the specific online community by neutral phrases in […] and precise texts are available upon request. Words in {} were adjusted to fit the specific person.

Hi ……….!
How are you? I'll introduce myself: My name is Floortje, I'm 24 years old and I'm from {country}. I love to travel, and I'm an outdoorsy! I'm pretty new to [this community], but I have [provided the service] a number of times already, as you can see on my profile. I really like to meet people through [community] experiences. Pretty soon I'll be traveling to your city and I'm really excited. I'd love to [use your service] during my stay there. I'll arrive May 22 and leave around May 26. Do you think you could [provide the service to me]? It's ok if you can't [provide it every day]. I hope to hear from you, and I hope we will meet shortly!
Thank you in advance and best regards,
{Name}

Hi ……….!
How are you? I'll introduce myself: My name is Floor, I'm 24 years old and I'm from {country}. I love to travel, and I'm an outdoorsy! I'm pretty new to [this community], so I haven't [provided or received the service] yet, as you can see on my profile. I'd really like to meet people through [community] experiences. Pretty soon I'll be traveling to your city and I'm really excited. I'd love to [use your service] during my stay there. I'll arrive May 22 and leave around May 26. Do you think you could provide the service to me]? It's ok if you can't [provide it every day]. I hope to hear from you, and I hope we will meet shortly!
Thank you in advance and best regards,
(Name)
